# Characterization of human papillomavirus genotypes and their coverage in vaccine delivered to Ethiopian women

**DOI:** 10.1038/s41598-024-57085-z

**Published:** 2024-04-04

**Authors:** Alemayehu Abate, Abaineh Munshea, Endalkachew Nibret, Dawit Hailu Alemayehu, Ashenafi Alemu, Alemseged Abdissa, Adane Mihret, Markos Abebe, Andargachew Mulu

**Affiliations:** 1 Department of Health Biotechnology, Institute of Biotechnology, Bahir Dar University, P. O. Box 79, Bahir Dar, Ethiopia; 2 Amhara Public Health Institute, Bahir Dar, Ethiopia; 3 Department of Biology, College of Science, Bahir Dar University, P. O Box 79, Bahir Dar, Ethiopia; 4 Armauer Hansen Research Institute, Addis Ababa, Ethiopia

**Keywords:** Biotechnology, Cancer, Microbiology

## Abstract

Cervical cancer is a significant public health concern in Ethiopia. It is mainly caused by persistent infection with the human papillomaviruses. The aim of this study was to assess the relationship between carcinogenic risk of probable, possible and low risk HPV infection and those of cervical intraepithelial neoplasia (CIN) and cervical cancer. A cross sectional study nested from prospective cohort study was conducted in Bahir Dar, northwest Ethiopia. Statistical analyses were performed using SPSSversion 26.0. HPV-16 was associated with a relatively higher risk of CIN II^+^, (AOR = 15.42; 95% CI 6.81–34.91). In addition, HPV-52, -18, -53 and -58, were significantly associated with an increased risk of CIN II^+^, (AOR = 7.38 (1.73–31.54), 5.42 (1.61–18.31), 4.08 (1.53–10.87), and 3.17 (1.00–10.03)), respectively. The current study shows high rate of HPV with predominance of HPV-16, -53, -58, -18, -35, and -52. The quadrivalent and nonavalent vaccine had only covered 27.1% and 45% of the circulating HPV genotypes. Ethiopia may need to consider introduction of nonavalent vaccine into the national public health strategy. Polyvalent vaccine which includes the genotypes not covered by existing approved vaccines should be considered.

## Introduction

Human papillomaviruses (HPVs) are the most common sexually transmitted viruses comprising more than 230 fully characterized types, even though new HPV types are still being discovered^1^. The International Agency for Research on Cancer (IARC) classify HPV as follows: carcinogenic HPV subtypes (group 1); probable carcinogenic HPV subtypes (group 2A); possible carcinogenic HPV subtypes (group 2B); low risk HPV subtypes (group 3) and those not classified with respect to carcinogenic potential^2^. The highest potential for cancer is posed by HPV-16, whereas HPV-18, -31, -33, -35, -39, -45, -51, -52, -56, -58, -59 categorized as human carcinogenic types, HPV-68 as probable carcinogenic, HPV-26, -30, -34, -53, -66, -67, -69, -70, -73, -82, -85, -97 as possible carcinogenic^3^, and HPV-6, -11, -42, -43, and -44 as low-risk HPV genotypes^4^.

Although the majority of infections are resolved by the host's immune system within a period of about two years, approximately 10% of HPV infection will persist in affected individuals^5^. Invasive cervical cancer and cervical intraepithelial neoplasia (CIN) are primarily associated with human papillomaviruses (HPVs), which serve as the main risk factor. The impact of specific HPV types on cervical cancer incidence can differ across locations, likely attributable to geographic variations in the prevalence of HPV types within different populations. The likelihood of progression from HPV infection to cervical cancer can also vary depending on the HPV genotype^6^.

Cervical cancer is the second most common cancer, next to breast cancer among women worldwide. Despite being one of the most preventable tumors among all major human cancers, the frequency of new cases of cervical cancer is rising steadily^7^.

Cervical cancer is a significant public health concern in Ethiopia, particularly among women between the ages of 15 and 44^8^. It is the most frequent malignancy affecting this population. Recent estimates indicate that approximately 7445 new cases of cervical cancer are diagnosed annually in the country, leading to the loss of 5338 women’s lives each year^9^.

Epidemiological studies are crucial for screening and immunization programs in addition to providing a thorough understanding and assessment of the risk of Cervical Cancer (CC) development in women infected with HR-HPV types^10^. The 2-valent HPV vaccine (Cervarix, 2vHPV), 4-valent HPV vaccine (Gardasil, 4vHPV), and 9-valent HPV vaccine (Gardasil 9, 9vHPV) are the three licensed HPV vaccinations that are currently available to prevent cervical cancer^11^. HPV-16 and -18, the two main kinds of HPV that cause cancer, HPV-6 and HPV-11, are protected against by the 2- and 4-valent vaccines; additionally, the 9-valent vaccine offers protection against HPV-31, -33, -45, -52, and -58^6^.

Since the current vaccines, particularly a quadrivalent vaccine given in Ethiopia, only provide a limited amount of cross-protection, gathering scientific data on the prevalence of HPV, genotype distribution, cytological profile, and related factors across various populations is crucial to forecasting the vaccine's effectiveness and developing new vaccination strategies^12^.

In this study, we assessed the association between carcinogenic risk of probable, possible and low risk HPV infection and those of cervical intraepithelial neoplasia (CIN) and cervical carcinoma.

## Results

A total of 297 women attending gynecology unit of Felege Hiwot Comprehensive Specialized Hospital were participated in the study. About 61.3% (182/297) of the participants live in rural area, majority of study participants (84.5%) were illiterates, more than half of study participants (63%) were married (Table 1).

**Table 1:** Socio-demographic, and economical characteristics of women attending gynecology unit, Felege Hiwot Comprehensive Specialized Hospital, Bahir Dar, northwest Ethiopia.

Variables	Categories	Total frequencies	Percentage (%)
Age (year)	< 30	28	9.4
	30–50	185	62.3
	> 50	84	28.3
Educational status	Illiterate	251	84.5
	Read and write	15	5.1
	Elementary	11	3.7
	High school and above	20	6.7
Monthly income	< 2000 birr	62	20.9
	2001–5000 birr	108	36.4
	> 5000 birr	127	42.8
Marital status	Single	44	14.8
	Married	187	63.0
	Divorced	27	9.1
	Widowed	39	13.1
Occupation	Employed	108	36.4
	Others	189	63.6
Residence	Rural	182	61.3
	Urban	115	38.7
HIV status	Positive	59	20
	Negative	238	80

### Pattern of HPV genotypes among the study population

The median age of study participants was 47 years (range: 25 to 85 years). The total prevalence of any HPV types was 46.8% (139/297), single infection and multiple (two or more) infection accounted 34.01% (101/297) and 12.79% (38/297), respectively. The rates of high risk and probable HR HPV genotypes was 34.68% (103/297); single infection and multiple infection accounting 23.23% (69/297) and 11.45% (34/297), respectively. The rates of possible HR and low risk HPV only was 12.12% (36/297) with 9.76% (29/297) single infection and 2.36% (7/297) multiple infection (Table 2).

**Table 2:** Characteristics of the study population among women attended cervical unit of FHCSH from January and December 2023.

HPV types	Total (N = 297)	Pathological grade			
		CIN II^+^ (N = 56)	CIN II^−^ (N = 241)	CIN I (N = 29)	Negative (N = 212)
High risk and probable HR HPV	103 (34.68%)	47 (83.93%)	56 (23.23%)	24 (82.76%)	32 (15.09%)
Type-16	25 (24.27%)	16 (28.57%)	9 (3.73%)	3 (10.34%)	6 (2.83%)
Type-18	11 (10.68%)	4 (7.14%)	7 (2.90%)	4 (13.79%)	3 (1.42%)
Type-16 and 18	2 (1.94%)	2 (3.57%)	0 (0.0%)	0 (0.0%)	0 (0.0%)
Type-16 and other probable	15 (14.56%)	7 (12.5%)	8 (3.32%)	5 (17.24%)	3 (1.42%)
Type-16, 18 and other probable	1 (0.97%)	1 (1.78%)	0 (0.0%)	0 (0.0%)	0 (0.0%)
Type-18 and other probable	3 (2.91%)	2 (3.57%)	1 (0.4%)	1 (3.45%)	0 (0.0%)
Type-other probable	46 (44.66%)	15 (26.78%)	31 (12.86%)	11 (37.93%)	20 (9.43%)
Possible HR HPV	36 (12.12%)	7 (12.5%)	29 (12.03%)	5 (17.24%)	24 (11.32%)
Type-53 alone	11 (30.55%)	2 (3.57%)	9 ( (3.73%)	3 (10.34%)	6 (2.83%)
Type-53 and other possible	4 (11.11%)	3 (5.35%)	1 (0.4%)	0 (0.0%)	1 (0.47%)
Type-70 alone	5 (13.89%)	2 (3.57%)	3 (1.24%)	1 (3.45%)	2 (0.94%)
Type-73 alone	2 (5.56%)	0 (0.0%)	2 (0.83%)	1 (3.45%)	1 (0.47%)
Other possible and low risk	14 (38.89%)	0 (0.0%)	14 (5.81%)	0 (0.0%)	14 (6.60%)
Negative	158 (53.19%)	2 (3.57%)	156 (64.73%)	0 (0.0%)	156 (64.73%)

### Distribution of HPV genotypes

In this study, at least one HPV genotype was detected in 139 (46.8%) of the total 297 samples investigated in this study. The HPV-16 genotype was the most frequent genotype, detected in 43/297 (14.48%) and the most common HPV type in patients with CIN II^+^ 26/56 (46.43%) of the patients followed by HPV-18, HPV-58, HPV-52, and HPV-35 infections, accounting for 9 (16.1%), 9 (16.1%), 7 (12.5%), and 6 (10.7%), respectively. Among patients with CIN II^−^, HPV-16 was the most common type, followed by HPV-58, HPV-53, HPV-18, and HPV-35 (Table 3).

**Table 3:** Individual occurrence of HPV according to histological examination among women attended cervical unit of FHCSH from January and December 2023.

HPV type	Individual occurrence (N = 297)	Pathological grade			
		CIN II^+^ (N = 56)	CIN II^−^ (N = 241)	CIN I (N = 29)	Negative (N = 212)
Type-16	43 (14.48%)	26 (46.4%)	17 (7.05%)	8 (27.59%)	9 (4.25%)
Type-18	17 (5.72%)	9 (16.1%)	8 (3.32%)	5 (17.24%)	3 (1.42%)
Type-31	2 (0.67%)	1 (1.8%)	1 (0.41%)	1 (3.45%)	0 (0.0%)
Type-33	5 (1.68%)	2 (3.6%)	3 (1.24%)	0 (0.0%)	3 (1.42%)
Type-35	14 (4.71%)	6 (10.7%)	8 (3.32%)	2 (6.90%)	6 (2.83%)
Type-39	2 (0.67%)	0 (0.0%)	2 (0.83%)	0 (0.0%)	2 (0.94%)
Type-45	3 (1.01%)	1 (1.8%)	2 (0.83%)	0 (0.0%)	2 (0.94%)
Type-51	4 (1.35%)	1 (1.8%)	3 (1.24%)	1 (3.45%)	2 (0.94%)
Type-52	12 (4.04%)	7 (12.5%)	5 (2.07%)	2 (6.90%)	3 (1.42%)
Type-56	5 (1.68%)	1 (1.8%)	4 (1.66%)	1 (3.45%)	3 (1.42%)
Type-58	21 (7.07%)	9 (16.1%)	12 (4.98%)	5 (17.24%)	7 (3.30%)
Type-59	2 (0.67%)	1 (1.8%)	1 (0.41%)	1 (3.45%)	0 (0.0%)
Type-68	8 (2.69%)	3 (5.4%)	5 (2.07%)	3 (10.34%)	2 (0.94%)
Type-66	7 (2.36%)	1 (1.8%)	6 (2.49%)	1 (3.45%)	5 (2.36%)
Type-6	4 (1.35%)	0 (0.0%)	4 (1.66%)	0 (0.0%)	4 (1.89%)
Type-11	1 (0.34%)	0 (0.0%)	1 (0.41%)	0 (0.0%)	1 (0.47%)
Type-26	2 (0.67%)	0 (0.0%)	2 (0.83%)	0 (0.0%)	2 (0.94%)
Type-40	6 (2.02%)	0 (0.0%)	6 (2.49%)	1 (3.45%)	5 (2.36%)
Type-42	6 (2.02%)	1 (1.8%)	5 (2.07%)	1 (3.45%)	4 (1.89%)
Type-43	2 (0.67%)	0 (0.0%)	2 (0.83%)	0 (0.0%)	2 (0.94%)
Type-44	8 (2.69%)	1 (1.8%)	7 (2.90%)	2 (6.90%)	5 (2.36%)
Type-53*	31 (10.44%)	12 (21.4%)	19 (7.88%)	7 (24.14%)	11 (5.19%)
Type-54	7 (2.36%)	0 (0.0%)	7 (2.90%)	1 (3.45%)	6 (2.83%)
Type-61	7 (2.36%)	0 (0.0%)	7 (2.90%)	0 (0.0%)	7 (3.30%)
Type-70**	17 (5.72%)	6 (10.7%)	11 (4.56%)	4 (13.79%)	7 (3.30%)
Type-73	2 (0.67%)	0 (0.0%)	2 (0.83%)	1 (3.45%)	1 (0.47%)
Type-82	2 (o.67%)	0 (0.0%)	2 (0.83%)	0 (0.0%)	2 (0.94%)

*16 of Type 53 occur with Probable HPV, **8 of Type 70 occur with probable HPV.

### Carcinogenic risk of individual HPV types

From the total number of participants, 56 (18.9%) of them had histo-pathological positive results. Majority, 54/56 (96.43%) of CIN II^+^ had HPV infection and 2/56 (3.57%) did not have HPV infection. This could be due to the HPV genotypes that could not be detected in the method. To assess the association of individual HPV types with CIN II^+^, according to the individual HPV types, we estimated the crude odds ratio (COR) and adjusted odds ratio (AOR) of having CIN II^+^ compared to CIN II^−^.

HPV-16 was associated with the highest risk of CIN II^+^, (AOR = 15.42; 95% CI 6.81–34.91). In addition, HPV-52, -18, -53 and -58, were significantly associated with an increased risk of CIN II^+^, (AOR = 7.38 (1.73–31.54), 5.42 (1.61–18.31), 4.08 (1.53–10.87), and 3.17 (1.00–10.03)) respectively and these data are summarized in Tables 4 and 5.

**Table 4:** Bivariate logistic regression analysis of HPV genotypes according to histological examination among among women attended cervical unit of FHCSH from January and December 2023.

HPV types	Individual frequencies	CIN II^+^ (n = 56)	CIN II^−^ (n = 241)	COR (95% CI)	P-value
Type-16	43	26	17	11.42 (5.56–23.47)	< 0.05
Type-18	17	9	8	5.58 (2.05–15.20)	< 0.05
Type-31	2	1	1	4.36 (0.27–70.85)	0.300
Type-33	5	2	3	2.94 (0.48–18.01)	0.244
Type-35	14	6	8	3.49 (1.16–10.52)	< 0.05
Type-45	3	1	2	2.17 (0.19–24.39)	0.529
Type-51	4	1	3	1.44 (0.15–14.13)	0.753
Type-52	12	7	5	6.74 (2.06–22.12)	< 0.05
Type-56	5	1	4	1.08 (0.12–9.83)	0.947
Type-58	21	9	12	3.65 (1.46–9.17)	< 0.05
Type-59	2	1	1	4.36 (0.27–70.85)	0.300
Type-68	8	3	5	2.67 (0.62–11.53)	0.188
Type-66	7	1	6	0.71 (0.08–6.04)	0.76
Type-42	6	1	5	0.89 (0.09–7.49)	0.89
Type-44	8	2	6	1.45 (0.29–7.39)	0.65
Type-61	7	1	6	0.71 (0.08–6.04)	0.76
Type-53	31	12	19	3.19 (1.44–7.03)	< 0.05
Type-70	17	6	11	2.51 (0.89–7.10)	0.083

*Type-39, Type-6, Type-11, Type-26, Type-40, Type-43, Type-54, Type-73 and Type-82 did not have participation in disease development (CIN II^+^ cases).

**Table 5:** Multivariate logistic regression analysis of HPV genotypes according to histological examination among women attended cervical unit of FHCSH from January and December 2023.

HPV types	Individual frequencies	CIN II^+^ (n = 56)	CIN II^−^ (n = 241)	AOR (95% CI)	P-value
Type-16	43	26	17	15.42 (6.81–34.91)	< 0.05
Type-18	17	9	8	5.42 (1.61–18.31)	< 0.05
Type-35	14	6	8	2.77 (0.76–10.16)	0.124
Type-52	12	7	5	7.38 (1.73–31.54)	< 0.05
Type-58	21	9	12	3.17 (1.00–10.03)	< 0.05
Type-53	31	12	19	4.08 (1.53–10.87)	< 0.05

### Vaccine coverage rate of detected HPVs

A total of 28 HPV genotypes were detected using Anyplex™ II HPV28 Detection system. The number of individual HPV types identified in this study was 240, only 45% of the HPVs genotypes included in the nonavalent vaccine (HPV -6, -11, -16, -18, -31, -33, -45, -52, and -58). The HPVs genotypes which are included in quadrivalent (HPV-6, -11, -16, -18) vaccine had a proportion of 27.1% and the quadrivalent (HPV-6, -11, -16, -18) vaccine had only covered 27.1% of the circulating genotypes. The bivalent vaccine (HPV-16/18) which is currently available in Ethiopia only covered 25% of the genotypes. But some genotypes with oncogenic risks are not covered by these licensed vaccines and are unfortunately found in our populations. The genotypes not covered by available HPV vaccine had proportion 55% (Table 6).

**Table 6:** Coverage rate of HPV genotypes by HPV vaccine among women attended cervical unit of FHCSH from January and December 2023.

HPV genotypes	Vaccine type	Frequency	Percentage (%)
6/11/16/18/31/33/45/52/58	Nonavalent	108	45
6/11/16/18	Quadrivalent	65	27.1
16/18	Bivalent	60	25
35/39/51/56/59/66/68/26/40/42/43/44/53/54/61/70/73/82	Not covered	132	55

## Discussion

The results of this study revealed that 139 (46.8%) of the study participants had an overall prevalence of HPV. This finding was in line with the results of another Ethiopian study that found 53.0% of women with cervical abnormalities being infected with HPV^13^. This finding is also consistent with research conducted in South Africa, where the total prevalence of HR-HPV DNA was 48.1%^14^, and Europe, where the study population’s overall HPV prevalence was reported to be 50.8%^15^.

Our finding indicated overall, there were 56 (18.85%) study participants with CIN II^+^ (classified as diseased) with pathology examination. Of CIN II^+^, 54/56 (96.43%) had HPV infection whereas two out of fifty six (3.57%) did not have HPV infection. The prevalence of HPV among women with CIN II^+^ was higher compared to women with CIN II^−^. This finding is similar with another study conducted in South Africa which detected HPV infection in 84.2% (383/455) of women^16^.

This result is also in line with a Chinese study that found women with abnormal cervical lesions and cervical cancer as having high HPV prevalence compared to women with normal cervical histology 10.5% for the Negative for Intraepithelial Lesion or Malignancy (NILM), 73.9% for CIN1, 89.5% for CIN2, and 91.5% for ≥ CIN3^17^. The study conducted in India also reported the prevalence of high risk HPV infection to be 93.3% in participants with invasive cervical cancer and 46.1% in those with precancerous lesions, indicating that HPV is a significant risk factor for cervical cancer^18^.

In our study, HPV-16 genotype, which was detected in 43 cases (14.48%), was the most common genotype. Its frequency rises as cervical cancer becomes more severe. The second most common genotype was HPV-58, which accounted for 21 (7.07%). HPV-18, -35, and -52 were the next in descending order accounting for 17 (5.72%), 14 (4.71%), and 12 (4.04%), respectively. This result has similar finding with study done in Ethiopia, which found that HPV-16 (37.3%), HPV-52 (6.8%), HPV-35 (4.8%), HPV-18 (4.4%), and HPV-56 (3.9%) were the most frequently detected genotypes^19^.

HPV-16 is the most prevalent genotype that is regularly mentioned as a major contributor to cervical abnormalities worldwide A systematic review study indicated that the pooled prevalence of the five most prevalent high-risk HPV types in Africa were HPV-16 (35.3%), HPV-52 (14.2%), HPV-35 (12.4%), HPV-18 (10.4%), and HPV-58 (10.0%)^20^. A different result is observed from a study done in Togo which reported HPV-16 as the least common genotype even though HPV-16 is the most frequent genotype in the world, according to this study the most prevalent genotypes were HPV-56 (22.7%), followed by HPV-51 (20.3%), HPV-31 (19.5%), HPV-52 (18.8%) and HPV-35 (17.2%)^21^.

The distribution of HPV genotypes is different in different parts of the world and among different studies^22–28^. The heterogeneity observed in the HPV genotype distribution among the studies could be attributed to variations in the spread of the HPV virus, sociodemographic composition of the study population, the degree of cervical lesions, and the diagnostic techniques used when examining women with abnormalities.

In the present study, the most common HPV type in patients with CIN II^+^ was HPV-16 which accounts 26 (46.4%), followed by HPV-53, HPV-58, HPV-18, HPV-52, and HPV-35 infections, accounting for 9 (16.1%), 9 (16.1%), 7 (12.5%), and 6 (10.7%) respectively. Infection by a specific type of HPV significantly increases the chance of developing cervical abnormalities. The risk that an HPV infection will progress to cervical cancer varies according to the HPV genotype. Among patients with CIN II^−^, HPV-16 was the most common type, followed by HPV-58, HPV-53, HPV-18, and HPV-35. HPV-16 was associated with the highest risk of CIN II^+^, (AOR = 15.42; 95% CI 6.81–34.91). In addition, HPV-52, -18, -53, and -58, in descending order, were significantly associated with an increased risk of CIN II^+^, (AOR = 7.38 (1.73–31.54), 5.42 (1.61–18.31), 4.08 (1.53–10.87), and 3.17 (1.00–10.03)) respectively.

Our results are supported by a Korean study, which showed that HPV-16 was linked to the highest risk of high grade squamous intraepithelial lesion and carcinoma (OR = 11.75; 95% CI 8.55–16.15). Furthermore, there was a significant correlation between an elevated risk of HSIL+ and HPV-33, -31, -52, -18, -58, -51, and -35 (OR ranging from 3.50 [HPV-33] to 2.62 [HPV-35])^6^. A study conducted in China which supports our finding reported HPV-16, -31, -33 and -58 were found to have considerably greater infection rates in patients with HSIL and higher lesions^29^.

The most significant approach both to prevent and eradicate cervical cancer is vaccination. The U.S. Food and Drug Administration (FDA) has approved three HPV vaccines: the bivalent HPV vaccine (Cervarix, 2vHPV), the quadrivalent HPV vaccine (Gardasil, 4vHPV), and the nonavalent HPV vaccine (Gardasil 9, 9vHPV). Ethiopia launched the quadrivalent vaccination for the first time in December 2018 targeting 14-year-old girls, with the support of the Global Alliance for Vaccine and Immunization (GAVI)^30^. The results of our study showed that 25% of the genotypes were covered by the bivalent vaccine (HPV-16/18). The quadrivalent (HPV-6, -11, -16, and -18) vaccination had a coverage rate of 27.1% and the nonavalent vaccine had a coverage rate of 45%. In line with our findings, a systematic review conducted among women in West Africa, found that 15.1% of the genotypes identified were covered by the bivalent vaccine (HPV-16/18). Furthermore, the nonavalent vaccine's additional high-risk carcinogenic HPVs (HPV-31/33/45/52/58) showed a prevalence of 37.6%. Thus, the nonavalent vaccine had a coverage rate of 55.8%. The prevalence of the HPV-35/39/51/56/59/66/68 genotypes, which are not protected by a vaccine, was 44.2%^30^.

The findings of the current study was also similar with a study conducted in Korea, which found that 12.0% of HPV-positive patients had HPV-16 and -18, which are protected against by all HPV vaccines. HPV-31, -33, -45, -52, and -58, which are covered by the 9-valent vaccine, accounted for 19.8% of HPV-positive patients. The remaining HR types (31.7%) among HPV-positive individuals were HPV-35, -39, -51, -56, -59, -66, and -68^6^. The 9-valent vaccine may have a significant impact on the prevention of cervical cancer in Ethiopia; nevertheless, for Ethiopian women, a polyvalent vaccine that protects against more prevalent genotypes (HPV-16/18/35/52/58/53) that cause cervical cancer is necessary.

In our study, the proportion of HPV genotypes non-covered by the existing vaccine was 132/240 (55%). This finding was consistent with a study conducted in Ethiopia that reported the prevalence of non‑vaccine‑targeted HPV was 56 (51.8%, 95% CI 0.42, 0.61)^31^.

## Conclusion

The prevalence of HPV among Ethiopian women was found to be high with predominance of HPV-16, -53, -58, -18, -35 and, -52. HPV-16, HPV-52, HPV-18, HPV-53, and HPV-58 were associated with the highest risk of CIN II^+^. In our study the quadrivalent and nonavalent vaccine had 27.1% and 45% coverage. Ethiopia may need to consider introduction of nonavalent vaccine into the national public health strategy as part of a comprehensive approach to cervical cancer prevention and control. Polyvalent vaccine which includes the genotypes not covered by existing vaccines should be considered to prevent the spread of HPV and cervical cancer.

## Methods

### Study setting and population

A cross sectional study nested from prospective cohort study was conducted between January and December 2023 among patients attending the gynecology unit of Felege Hiwot Comprehensive Specialized Hospital (FHCSH) in Bahir Dar, northwestern Ethiopia. A total of 324 women of age above 30 and HIV positive women with all age range (according to WHO recommendation for screening) who visited FHCSH Gynecological unit who come for gynecological complaints such as vaginal bleeding, vaginal discharge, abdominal pain, back pain, Difficulty of urination, difficulty of defecation, protruded mass per vagina, burning sensation and itching, pain during sexual intercourse and urination during data collection time were enrolled, but 27 of them had insufficient PAP smears and they were excluded from the study, the rest 297 were included and all gave their informed consent to participate in the study. All women who visit FHCSH gynecological unit, above 30 years old and willing to participate in the study were included. Although the age of HIV positive women was less than 30, they were included in the study. All women who took any kind of treatment for cervical cancer or any vaginal medication, vaginal contraceptives or douches 48 h prior to the test, those who had sexual intercourse 24 h before the test; women who are in menstruation, pregnant and women who had hysterectomy were excluded from the study.

All of the study participants were screened with Visual acetic acid test (VIA), genotyping of the HPV DNA test and cervical cytology examination with the PAP test. Study participants with positive results from screening tests were examined with colposcopy and biopsy was taken for histopathology.

### Source population

The study population was all women of age above 30 and HIV positive women with all age range who visited FHCSH Gynecological unit during data collection time.

### Study population

The study population was all women of age above 30 and HIV positive women with all age range who visited FHCSH Gynecological unit and suspected for cervical cancer.

### Sample size determination

Study one: A single population proportion sample size determination formula (study population size less than 10,000^32^) was used with the assumption of 19% proportion of HPV, 5% margin of error, and 95% desired level of confidence interval and considering a 10% non-response rate, the sample size was 281.

Study two: Sample size was determined from formulation of sensitivity and specificity test using Power Analysis and Sample Size (PASS) software based on desired type I error, power and effect size^33^. The minimum sample size was determined by taking the prevalence of a disease 19%, by assuming sensitivity of the kit is comparable with the gold standard, and specificity of the kit is greater than 70%, the power is set to be at least 80% and the P-value, is set to be less than 0.05. The sample size for sensitivity and specificity of Onco E6 performance study was 49 positive for histopathology, 196 negative samples with the gold standard histopathology examination and 49 negative controls. By taking 10% contingency, the sample size was 324. The final sample size was the highest sample size of the upper studies, 324 sample size was determined for this study.

### Sampling technique and procedure

The sampling technique was systematic random sampling. Every third study participants was selected after a random starting study participant who was selected by lottery method.

### Follow up visits

All study participants were screened with Visual acetic acid test (VIA), genotyping of the HPV DNA test and cervical cytology examination with PAP test. Study participants with positive results from screening tests were examined with colposcopy and biopsy was taken for histopathology. Participants with positive results of the screening or diagnostic tests were followed. Two follow-up visits were scheduled 6 and 12 months after the baseline visit.

### Specimen collection

Trained gynecologists, working in the cervical cancer unit, carried out gynecological and physical examination services to the participants. They conducted visual inspection with acetic acid (VIA) screenings and collected swab specimens for HPV DNAtesting, as well as cytologic specimens and biopsies in cases where there were positive results from colposcopic impressions. The swab specimens were collected using PreservCyt solution from Halogic.Inc. following the manufacturer’s instructions for the collection and handling of cervical specimens in PreservCyt solution. All the collected cervical swabs were stored at − 80 °C at the Amhara Public Health Institute (APHI), Bahir Dar and later transported to the Armauer Hansen Research Institute (AHRI), Addis Ababa, for genotyping purposes.

### HPV genotyping and histopathology examinations

HPV genotyping was performed to detect high-risk HPV DNA in cervical swabs. Anyplex™ II PCR System were used for detection of human papillomavirus-19 high-risk HPV types (-16, -18, -26, -31, -33, -35, -39, -45, -51, -52, -53, -56, -58, -59, -66, -68, -69, -73, -82) and 9 low-risk HPV types (-6, -11, -40, -42, -43, -44, -54, -61, -70) from cervical swab specimens.

DNA was isolated using the Abbott *m*2000sp. automated sample preparation system using magnetic particles to capture the nucleic acid (Abbott *m*Sample Preparation System_*DNA*_ for RealTi*m*e HR HPV; Wiesbaden, Germany). Five μl of extracted DNA was tested in a final reaction volume of 100 μl with 50 μl of the kit working master mix containing MgCl_2_, KCl, Ampli*Taq* Gold DNA polymerase, Uracil-N-Glycosylase, dATP, dCTP, dGTP, dUTP, dTTP, and biotinylated PGMY primers and β-globin primers GH20 and PC04, as per the manufacturer recommendations. The master mix was prepaired by mixing 5 μl 4× HPV28 A TOM or B TOM, 5 μl Enzyme and 5 μl RNase-free Water supplied by the manufacturer.

Anyplex™II HPV28 detection consists of two PCR reactions (A set and B set). A set is a multiplex assay that permits the simultaneous amplification of target DNA of 14 high risk human papillomaviruses. B set is a multiplex assay that permits the simultaneous amplification of target DNA of 5 high risk and 9 low-risk human papillomaviruses. Anyplex™ II HPV28 detection represents Seegene’s proprietary technologies and is based on a TOCE™ (Tagging Oligonucleotide Cleavage Extension) technology which makes it possible to detect multi-pathogens in a single fluorescence channel on real-time PCR instruments (CFX96TM Real-time PCR System (Bio-Rad)). The controls were run in parallel with the samples and the IC results were reported parallel to HPV results in a sample. The PCR cycle of HPV detection was End point-CMTA (Melt analysis of one time) with 50 cycles.

Biopsies were independently examined by two experienced pathologists. When the diagnosis differed between the two pathologists, the sample was reviewed by a third pathologist and consensus was reached. Histo-pathological diagnosis confirmed test results as negative for dysplasia/malignancy or Cervical Intraepithelial Neoplasia (CIN) as CIN I, CIN II, CIN III or cervical cancer cases. Histopathological examination results of CIN II, CIN III and invasive carcinoma (“CIN II^+^”) were taken as positive (Disease-positive) by gold standard definition. Histopathologic diagnoses of normal, chronic cervicitis and CIN I (“CIN II^−^”) were considered negative (Disease-negative).

### Statistical analysis

Statistical analyses were performed using SPSSversion 26.0. Descriptive statistics such as frequency and cross tabulation were performed to summarize the data. Bivariate and multivariate logistic regression analysis was performed to test the relationship between HPV infection and severity of histological examinations. Crude Odds Ratio (COR) and Adjusted Odds Ratio (AOR) at 95% confidence interval were calculated to assess the degree of association between the odds of being in the CIN II^+^ group in the presence of an individual HPV type and the odds of being in the CIN II^+^ group in the absence of an individual HPV type (Considered as reference category in logistic regression). Finally, variables with a P-value of less than 0.05 were considered statistically significant.

### Ethics approval and consent to participate

Ethical approval was obtained from Institutional Review Board (IRB), Bahir Dar University. Written informed consent was ensured from all study participants to take part in the study voluntarily after they get informed about the objective and purpose of the study. This study was performed in accordance with the Declaration of Helsinki.

## Data Availability

All the generated data in this article are included in the manuscript. The original data can be obtained from the principal investigator upon request Alemayehu Abate alexu2love@gmail.com.

## Abbreviations

DNA: Deoxyribonucleic acid
HPV: Human papilloma virus
HR-HPV: High-risk human papilloma virus
CI: Confidence interval
STIs: Sexually transmitted infections
CIN: Cervical intraepithelial neoplasia
ICC: Invasive cervical cancer
PCR: Polymerase chain reaction
